# Challenging Glaucomas: Update on Diagnosis and Management

**DOI:** 10.1155/2016/6935086

**Published:** 2016-02-17

**Authors:** Gianluca Scuderi, Peng T. Khaw, Felipe A. Medeiros, Gianluca Manni

**Affiliations:** ^1^Sapienza University of Rome, Via di Grottarossa No. 1039, 00189 Rome, Italy; ^2^National Institute Health Research (NIHR), Biomedical Research Centre, Moorfields Eye Hospital NHS Foundation Trust and UCL Institute of Ophthalmology, London, UK; ^3^UC San Diego Shiley Eye Center, San Diego, CA, USA; ^4^Tor Vergata University of Rome, Viale Oxford 8, 00133 Rome, Italy

This special issue is focused on the current approaches used to identify and manage particular groups of glaucoma that we define as challenging glaucomas, because of difficulties in either diagnosis or therapy.

Malignant glaucoma is a good example of difficulties in both diagnosis and management, and J. Foreman-Larkin et al. provide a complete overview on the clinical management of malignant glaucoma. Pseudoexfoliation is also typified by very high pressures at times and rapid damage to the optic nerve and P. Plateroti et al. focus their paper on pseudoexfoliation syndrome and pseudoexfoliative glaucoma with a review of the literature and updates on surgical management. Raised pressure and inflammation can be very challenging and D. Lewkowicz et al. cover the clinical outcome of hypertensive uveitis. Infection after glaucoma surgery is one of the most challenging situations and K. Ohtomo et al. report their outcomes of late-onset bleb related endophthalmitis treated with pars plana vitrectomy.

The aetiology of different variants of the glaucoma with higher and lower ranges of pressure still poses a challenge to us. D. Wróbel-Dudzińska et al. describe the risk factors in normal-tension glaucoma and high-tension glaucoma in relation to polymorphisms of the endothelin-1 gene and the endothelin-1 receptor type A gene. Vascular changes may play a part in the pathogenesis of glaucoma, and E. C. Koch et al. discuss blood pressure and heart rate variability in the detection of vascular dysregulation in glaucoma.

Assessment of the cellular layers that get damaged may provide further clues in the assessment and diagnosis, and A. Perdicchi et al. present a research article on the evaluation of agreement between HRT 3 and I-Vue OCT in glaucoma and ocular hypertension patients comparing different tomographic approaches to evaluate retinal ganglion cells.

As the variety of topics presented in this special issue suggests, there are still many difficulties related to the diagnosis and therapies for glaucoma. This pathology, especially in its most advanced stages, can have a real impact on the quality of life and outlook of patients. L. Zuo et al. write about vision health-related quality of life in Chinese glaucoma patients, describing how glaucoma can influence the quality of life in patients.

There are some particular forms of glaucoma, which more than others, represent a challenge for the ophthalmologist. For example, pigmentary glaucoma has many grey areas that still need to be clarified, including its pathogenesis and the involvement of retinal structures. As mentioned, pseudoexfoliative glaucoma also presents many difficulties for both clinical and therapeutic approaches. However, paediatric forms of glaucoma represent one of the greatest clinical challenges, due to the variation in diagnostic criteria as well as in potential treatments and the response of individual patients. This is especially the case with those forms of glaucoma secondary to other congenital diseases of the anterior segment of the eye, particularly if associated with facial anomalies and other malformations of intraocular structures. This group of disorders is particularly complex for the ophthalmologist, with obstacles in the management of these patients from diagnosis to treatment.

However, daunting these challenges, the future looks encouraging as recent advances in translational research are reshaping modern ophthalmology including whole genome analysis and understanding of epigenetic influences and bioinformatics. This should allow the gradual identification of the link between different clinical phenotypes and specific mutation in genes regulating the normal formation and maturation of the eye, which should ultimately give rise to new therapeutic hypotheses and approaches.

Thanks to nanotechnologies, new methods of drug administration and the controlled release of molecules used to decrease intraocular pressure are being developed. Surgical treatments are also moving forward with new minimally invasive procedures and with other devices which show promising results with fewer side effects.

Furthermore, recent diagnostic criteria define glaucoma as a true neurodegenerative pathology, which affects not only the eye but also the whole optic tract and possibly involves the supportive cells of the central nervous system. This is the reason why, not only will future research focus on lowering IOP but also it will increasingly include therapies with neuroenhancing and neuroprotective effects and, in the most optimistic hypotheses, will lead to neuroregeneration.

*Gianluca Scuderi* *Gianluca Scuderi* *Peng T. Khaw* *Peng T. Khaw* *Felipe A. Medeiros* *Felipe A. Medeiros* *Gianluca Manni* *Gianluca Manni*

